# New Poly(imide)s Bearing Alkyl Side-Chains: A Study on the Impact of Size and Shape of Lateral Groups on Thermal, Mechanical, and Gas Transport Properties

**DOI:** 10.3390/membranes10070141

**Published:** 2020-07-04

**Authors:** Fidel E. Rodríguez-González, Germán Pérez, Vladimir Niebla, Ignacio Jessop, Rudy Martin-Trasanco, Deysma Coll, Pablo Ortiz, Manuel Aguilar-Vega, Luis H. Tagle, Claudio A. Terraza, Alain Tundidor-Camba

**Affiliations:** 1 Research Laboratory for Organic Polymers (RLOP), Department of Organic Chemistry, Pontificia Universidad Católica de Chile, Santiago 7810000, Chile; ferg@uc.cl (F.E.R.-G.); vniebla@uc.cl (V.N.); ltagle@uc.cl (L.H.T.); 2 Bureau Veritas Laboratories, 7150 Rue Frederick Banting, Saint-Laurent, Montreal, QC H4S 2A1, Canada; gmperez80@gmail.com; 3 Organic and Polymeric Materials Research Laboratory, Departamento de Química, Universidad de Tarapacá, Av. General Velásquez 1775, P.O. Box 7-D, Arica 1000000, Chile; iajessop@uta.cl; 4 Departamento de Química, Universidad Tecnológica Metropolitana, J. P. Alessandri 1242, Santiago 7810000, Chile; rudy.martint@utem.cl; 5 Núcleo de Química y Bioquímica, Facultad de Estudios Interdisciplinarios, Universidad Mayor, Santiago 3830000, Chile; deysma.coll@mayor.cl; 6 Centro de Nanotecnología Aplicada, Facultad de Ciencias, Universidad Mayor, Santiago 3830000, Chile; pablo.ortiza@mayor.cl; 7 Laboratorio de Membranas, Unidad de Materiales, Centro de Investigación Científica de Yucatán A.C. (CICY), Chuburna de Hidalgo, Merida, Yucatán 97205, Mexico; mjav@cicy.mx; 8 UC Energy Research Center, Pontificia Universidad Católica de Chile, Santiago 7810000, Chile

**Keywords:** aromatic poly(imide)s, bulky pendant groups, gas permeability, structure-property relationship

## Abstract

A set of five new aromatic poly(imide)s (PIs) incorporating pendant acyclic alkyl moieties were synthesized. The difference among them was the length and bulkiness of the pendant group, which comprises of linear alkyl chains from three to six carbon atoms, and a *tert*-butyl moiety. The effect of the side group length on the physical, thermal, mechanical, and gas transport properties was analyzed. All PIs exhibited low to moderate molecular weights (Mn ranged between 27.930–58.970 Da, and Mw ranged between 41.760–81.310 Da), good solubility in aprotic polar solvents, except for PI-*t*-4, which had a *tert*-butyl moiety and was soluble even in chloroform. This behaviour was probably due to the most significant bulkiness of the side group that increased the interchain distance, which was corroborated by the X-ray technique (**PI-*t*-4** showed two *d*-spacing values: 5.1 and 14.3 Å). Pure gas permeabilities for several gases were reported (**PI-3** (Barrer): He(52); H_2_(46); O_2_(5.4); N_2_(1.2); CH_4_(1.1); CO_2_(23); **PI-*t*-4** (Barrer): He(139); H_2_(136); O_2_(16.7); N_2_(3.3); CH_4_(2.3); CO_2_(75); **PI-5** (Barrer): He(44); H_2_(42); O_2_(5.9); N_2_(1.4); CH_4_(1.2); CO_2_(27); **PI-6** (Barrer): He(45); H_2_(43); O_2_(6.7); N_2_(1.7); CH_4_(1.7); CO_2_(32)). Consistent higher volume in the side group was shown to yield the highest gas permeability. All poly(imide)s exhibited high thermal stability with 10% weight loss degradation temperature between 448–468 °C and glass transition temperature between 240–270 °C. The values associated to the tensile strength (45–87 MPa), elongation at break (3.2–11.98%), and tensile modulus (1.43–2.19 GPa) were those expected for aromatic poly(imide)s.

## 1. Introduction

One of the most important technological advances at the end of the 20th century was the industrial development of processes that include polymeric membranes for water treatment and gas separation [1]. Membrane-based gas separation technologies have not yet succeeded in displacing traditional processes, such as separation by absorbents or cryogenic distillation, even when different polymeric materials have been tested as filters [2]. Therefore, numerous research teams continue to make progress to obtaining new polymers or mixtures of them, with very high permeability to gases [3,4,5,6].

Aromatic poly(imide)s have excellent thermal and mechanical properties, high resistance to chemical agents, and the ability to form thin layers films [7,8,9], qualities that make them suitable materials for gas separation. The poly(imide) Matrimid is one of the widely commercial polymers used in the preparation of membranes [10].

Many scientific papers have examined the relationship between the chemical structure of polymers and their macroscopic properties, including their behaviour as gas separating membranes [11,12,13,14]. Those studies have focused on changing the size and shape of the side groups to tune the stiffness of poly(imide)s main chain or to introduce functional groups that could be chemically modified post-polymerization. The use of monomers with contorting units that prevented efficient packaging of the chains incorporated bulky substituents close to imide groups to avoid the free rotation of the chain has also been tested. With those structural modifications, it is possible to modulate not only the permeability of the material but also properties such as solubility (processability), thermal stability, and mechanical resistance. For example, Huang et al. synthesized two novel diphenyl ether diamines with one or two *tert*-butyl groups as building blocks for the synthesis of new aromatic poly(imide)s. The *tert*-butyl groups increased the solubility of poly(imide)s due to the increase in the interchain distances [15]. Yao et al. studied the effect of attaching different side groups on the properties of poly(amide-imide)s, showing good solubility and excellent comprehensive properties [16]. Liou et al. prepared processable aromatic poly(imide) membranes containing trimethyl substituted triphenylamine units. The poly(imide)s were readily soluble in polar solvents and exhibited an improvement of gas permeability [17].

In previous works, we synthesized a new set of fluorinated aromatic poly(imide)s based on aromatic diamines with various cycloalkyl pendant groups and 4,4′-(hexafluoroisopropylidene)diphthalic anhydride (6FDA) as a common monomer for all poly(imide)s. Thermal, mechanical, and gas transport properties were studied to evaluate the impact of the volume of C3, C5, C6, C8, and C10 (adamantyl) cyclic lateral groups [18,19]. All poly(imide)s showed similar thermal resistance, reflecting the independency between their overall thermal stability and the bulkiness of the monocyclic pendant groups. Regarding mechanical resistance, the results suggested that the bigger the pendant group, the easier the materials deformed, and more tension was required to break them. Additionally, the polymer with the bulkiest pendant group (adamantyl) exhibited the largest interchain space, which was corroborated by X-rays, leading to the most permeable membrane.

Continuing with this previous work, here we present a set of new fluorinated aromatic poly(imide)s based on the same dianhydride (6FDA) and aromatic diamines with acyclic alkyl fragments as side groups formed by R-NHCO-chains of three, four, five, and six carbon atoms. A *tert*-butyl substituent was also used as R moiety to study the effect of the ramification against linearity. Their physical, thermal, mechanical, and pure gas transport properties were measured, and the results were compared among themselves and with those previously reported.

## 2. Materials and Methods

### 2.1. Materials

Butylamine (99 +%), pentylamine (99%), and hexylamine (99%) were purchased from Acros Organics (Morris Plains, NJ, USA). 3,5-dinitrobenzoyl chloride (DNBC) (96.5%), propylamine (98%), *tert*-butylamine (99.5%), 4,4′-(hexafluoroisopropylidene) diphthalic anhydride (6FDA) (99%), anhydrous *N*,*N*-dimethylacetamide (DMAc) (99.8%), triethylamine (TEA) (99.5%), hydrazine monohydrate (80%), anhydrous pyridine (99.8%), and Pd/C (10%) were purchased from Sigma-Aldrich (Milwaukee, WI, USA). All other reagents and solvents were purchased commercially as analytical grade and used without further purification.

### 2.2. Monomer Synthesis and Characterization

**Scheme 1:**
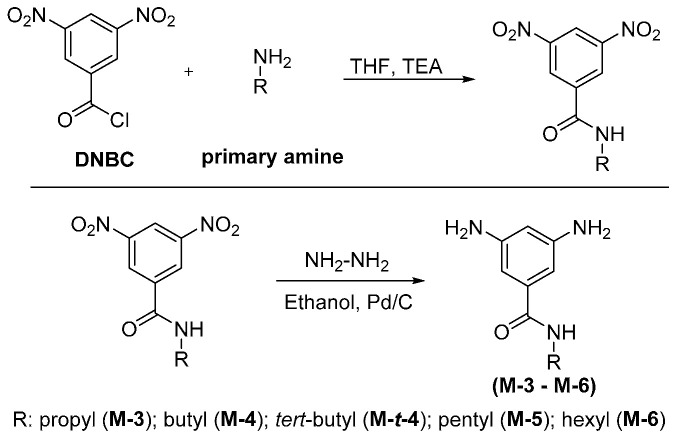
Synthesis of amide-diamine monomers.

Monomers were synthesized following the same procedure described in our previous paper [19]. Briefly, DNBC reacted with an excess of the corresponding amine (propylamine, butylamine, pentylamine, hexylamine, and *tert*-butylamine), using THF as solvent and TEA as base. The dinitro derivatives were obtained in yields between 86 and 89% (yield was calculated following the equation: Yield = (mol_exp_/mol_theorical_) × 100; Dinitro **M-3**: 86%; Dinitro **M-4**: 86%; Dinitro **M-*t*-4**: 89%; Dinitro **M-5**: 87% and Dinitro **M-6**: 86%). The 3,5-dinitro *N*-alkylbenzamide derivatives obtained were reduced to 3,5-diamine-*N*-alkylbenzamide derivatives by using monohydrate hydrazine (80%) in the presence of Pd/C and ethanol as solvent (Scheme 1). The amide-diamine monomers were purified by sublimation technique.

**M-3:** Yield: 93% (calculated in the same way for Dinitro derivatives). FT-IR (KBr, ν, cm^−1^): 3420, 3388 (N–H, amino); 3326 (N–H, amide), 3049 (C–H, arom.), 2955, 2926, 2855 (C–H, aliph.); 1645 (C=O); 1602, 1550, 1472 (C=C); 746 (out-of-plane ring bending). ^1^H NMR (400 MHz, DMSO-*d_6_*, δ, ppm) 7.76 (t, *J* = 5.2 Hz, 1H, H-7); 6.22 (s, 2H, H-4); 5.92 (s, 1H, H-1); 4.91 (broad peak, 4H, H-2); 3.22 (m, 2H, H-8); 1.51 (m, 2H, H-9); 0.87 (t, *J* = 7.2 Hz, 3H, H-10). ^13^C NMR (100 MHz, DMSO-*d_6_*, δ, ppm) 167.04 (C-6); 148.60 (C-3); 136.55 (C-5); 102.25 (C-4); 101.80 (C-1); 41.51 (C-8); 22.11 (C-9); 11.40 (C-10). Elem. Anal. Calcd. for C_10_H_15_N_3_O (193.25), C, 62.15%; H, 7.82%; N, 21.74%. Found: C, 62.09%; H, 7.93%; N, 21.65%.


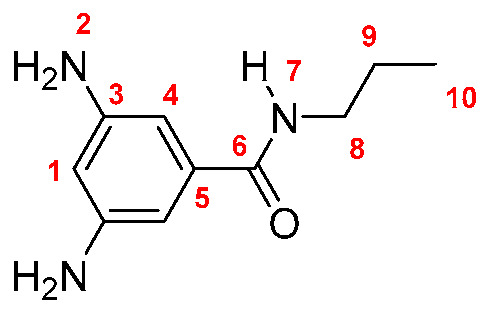


**M-4:** Yield: 92% (calculated in the same way for Dinitro derivatives). FT-IR (KBr, ν, cm^−1^): 3418, 3389 (N–H, amino); 3324 (N–H, amide), 3049 (C–H, arom.), 2959, 2920, 2855 (C–H, aliph.); 1646 (C=O); 1602, 1551, 1473 (C=C); 745 (out-of-plane ring bending). ^1^H NMR (400 MHz, DMSO-*d_6_*, δ, ppm) 7.75 (d, *J* = 5.0 Hz, 1H, H-7); 6.22 (s, 2H, H-4); 5.92 (s, 1H, H-1); 4.91 (broad peak, 4H, H-2); 3.25 (m, 2H, H-8); 1.49 (m, 2H, H-9); 1.33 (m, 2H; H-10); 0.86 (t, *J* = 7.0 Hz, 3H, H-11). ^13^C NMR (100 MHz, DMSO-d_6_, δ, ppm) 166.02 (C-6); 148.61 (C-3); 136.56 (C-5); 102.30 (C-4); 101.85 (C-1); 38.97 (C-8); 29.30 (C-9); 22.22 (C-10); 13.71 (C-11). Elem. Anal. Calcd. for C_11_H_17_N_3_O (207.28), C, 63.74%; H, 8.27%; N, 20.27%. Found: C, 62.99%; H, 8.43%; N, 19.95%.


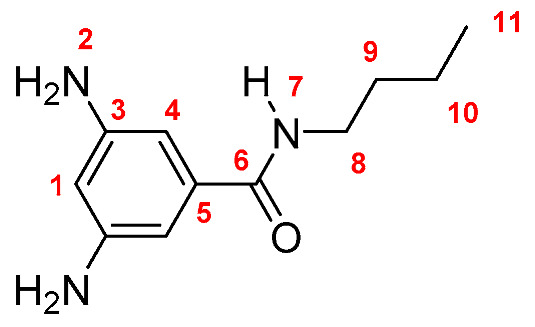


**M-*t*-4:** Yield: 92% (calculated in the same way for Dinitro derivatives). FT-IR (KBr, ν, cm^−1^): 3419, 3388 (N–H, amino); 3328 (N–H, amide), 3046 (C–H, arom.), 2959, 2865 (C–H, aliph.); 1646 (C=O); 1601, 1552, 1472 (C=C); 746 (out-of-plane ring bending). ^1^H NMR (400 MHz, DMSO-*d_6_*, δ, ppm) 6.92 (s, 1H, H-7); 6.20 (s, 2H, H-4); 5.88 (s, 1H, H-1); 4.90 (broad peak, 4H, H-2); 1.35 (s, 9H, H-9). ^13^C NMR (100 MHz, DMSO-*d_6_*, δ, ppm) 166.02 (C-6); 148.63 (C-3); 136.53 (C-5); 102.23 (C-4); 101.79 (C-1); 50.62 (C-8); 28.33 (C-9). Elem. Anal. Calcd. for C_11_H_17_N_3_O (207.28), C, 63.74%; H, 8.27%; N, 20.27%. Found: C, 63.17%; H, 7.95%; N, 20.03%.


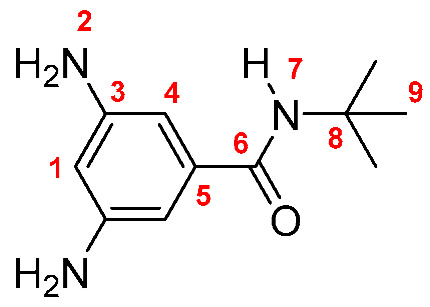


**M-5:** Yield: 87% (calculated in the same way for Dinitro derivatives). FT-IR (KBr, ν, cm^−1^): 3418, 3389 (N–H, amino); 3326 (N–H, amide), 3050 (C–H, arom.), 2959, 2920, 2855 (C–H, aliph.); 1647 (C=O); 1602, 1551, 1473 (C=C); 745 (out-of-plane ring bending). ^1^H NMR (400 MHz, DMSO-*d_6_*, δ, ppm) 7.74 (t, *J* = 5.1 Hz, 1H, H-7); 6.21 (s, 2H, H-4); 5.92 (s, 1H, H-1); 4.92 (broad peak, 4H, H-2); 3.23 (m, 2H, H-8); 1.51 (m, 2H, H-9); 1.29 (m, 4H; H-10, H-11); 0.85 (t, *J* = 6.6 Hz, 3H, H-12). ^13^C NMR (100 MHz, DMSO-*d_6_*, δ, ppm) 166.10 (C-6); 148.57 (C-3); 136.52 (C-5); 102.21 (C-4); 101.84 (C-1); 38.90 (C-8); 29.96 (C-10); 28.23 (C-9); 22.40 (C-11); 13.80 (C-12). Elem. Anal. Calcd. for C_12_H_19_N_3_O (221.30), C, 65.13%; H, 8.65%; N, 18.99%. Found: C, 64.89%; H, 8.43%; N, 18.23%.


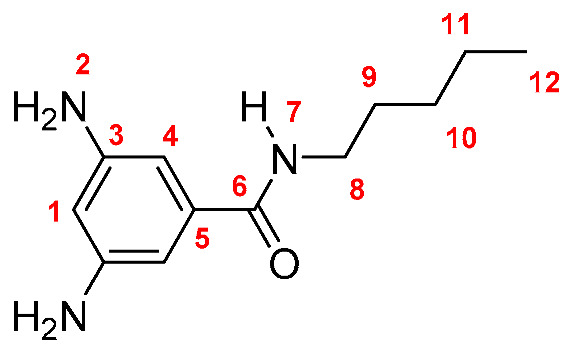


**M-6:** Yield: 91% (calculated in the same way for Dinitro derivatives). FT-IR (KBr, ν, cm^−1^): 3419, 3388 (N–H, amino); 3327 (N–H, amide), 3049 (C–H, arom.), 2959, 2921, 2857 (C–H, aliph.); 1647 (C=O); 1602, 1550, 1474 (C=C); 745 (out-of-plane ring bending). ^1^H NMR (400 MHz, DMSO-*d_6_*, δ, ppm) 7.74 (d, *J* = 5.2 Hz, 1H, H-7); 6.21 (s, 2H, H-4); 5.92 (s, 1H, H-1); 4.92 (broad peak, 4H, H-2); 3.23 (m, 2H, H-8); 1.50 (m, 2H, H-9); 1.26 (m, 6H; H-10, H-11, H-12); 0.84 (t, *J* = 6.7 Hz, 3H, H-13). ^13^C NMR (100 MHz, DMSO-*d_6_*, δ, ppm) 166.09 (C-6); 148.57 (C-3); 136.53 (C-5); 102.22 (C-4); 101.83 (C-1); 38.85 (C-8); 31.25 (C-11); 28.86 (C-9); 26.54 (C-10); 22.41 (C-12); 13.84 (C-13). Elem. Anal. Calcd. for C_13_H_21_N_3_O (235.33), C, 66.35%; H, 8.99%; N, 17.86%. Found: C, 66.00%; H, 8.27%; N, 17.15%.


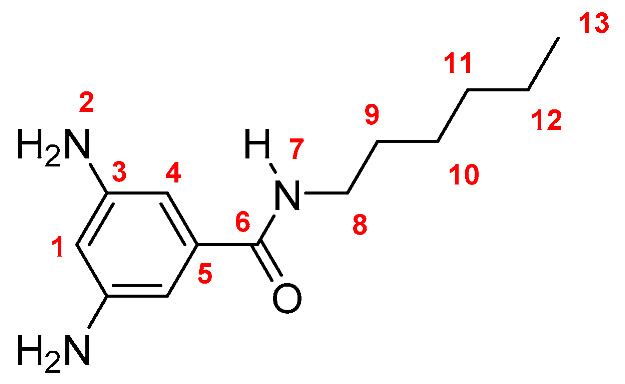


### 2.3. Polymer Synthesis and Characterization

**Scheme 2:**
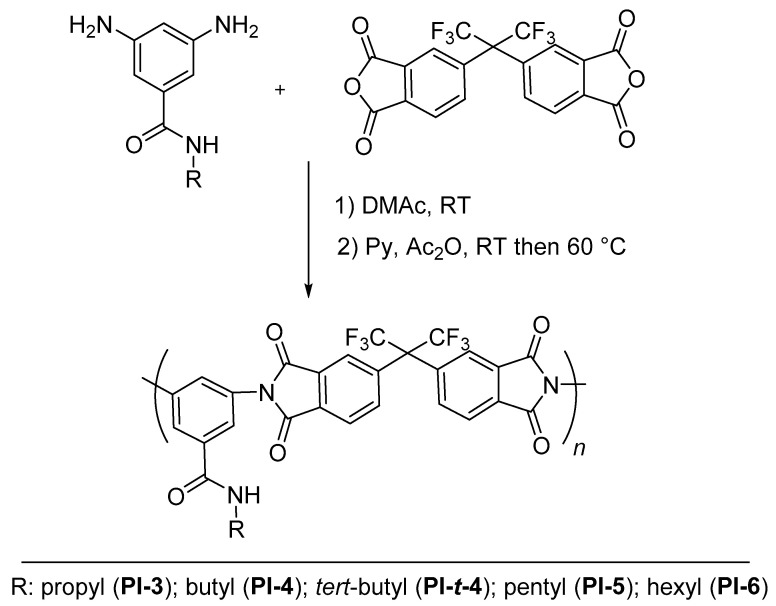
Synthesis of poly(imide)s.

A typical polymerization procedure for the synthesis of the poly(imide)s was followed (Scheme 2). To a three-necked round-bottomed flask equipped with mechanical stirrer and under the nitrogen atmosphere, a mixture of 2.0 mmol of 3,5-diamino-*N*-alkylbenzamide, 2.0 mmol 6FDA, and 4 mL of DMAc were added and stirred at room temperature for 6 h. After that, 1.0 mL of acetic anhydride and 0.8 mL of pyridine were added. The mixture was stirred for another two hours at room temperature and then raised and maintained at 60 °C for one more hour. Then, the mixture was cooled and poured in 300 mL of water under stirring. The white solid was filtered, thoroughly washed with methanol, and dried at 100 °C for 12 h.

**PI-3:** FT-IR (KBr, ν, cm^−1^): 3417 (N–H, amide); 3093 (C–H, arom.); 2965, 2928, 2875, 2855 (C–H, aliph.); 1785 (C=O asym., imide); 1729 (C=O sym., imide); 1659 (C=O, amide); 1597, 1536, 1457 (C=C, arom. ring); 1356 (C-N, imide); 721 (imide ring deformation). ^1^H NMR (600 MHz, DMSO-*d_6_*, δ, ppm) 8.59 (broad peak, 1H, H-16); 8.20 (d, *J* = 7.8 Hz, 2H, H-9); 8.04 (s, 2H, H-4); 7.98 (broad peak, 2H, H-10); 7.78 (s, 2H, H-13); 7.74 (s, 1H, H-11); 3.23 (broad peak, 2H, H-17); 1.52 (m, 2H, H-18); 0.87 (t, *J* = 7.2 Hz, 3H, H-19). ^13^C NMR (150 MHz, DMSO-*d_6_*, δ, ppm) 167.80 (C-15); 166.20 (C-7); 165.99 (C-6); 138.01 (C-3); 136.48 (C-5); 136.40 (C-14); 133.78 (C-10); 133.25 (C-12); 133.08 (C-8); 129.05 (C-11); 126.81 (C-13); 124.97 (C-9); 124.31 (C-4); 123.95 (q, *J* = 286 Hz, C-1); 65.05 (hept, *J* = 25 Hz, C-2); 40.70 (C-17); 21.89 (C-18); 11.72 (C-19). Elem. Anal. Calcd. for [C_29_H_17_F_6_N_3_O_5_]*_n_* (601.46)*_n_*, C, 57.91%; H, 2.85%; N, 6.99%. Found: C, 57.13%; H, 2.53%; N, 6.65%.


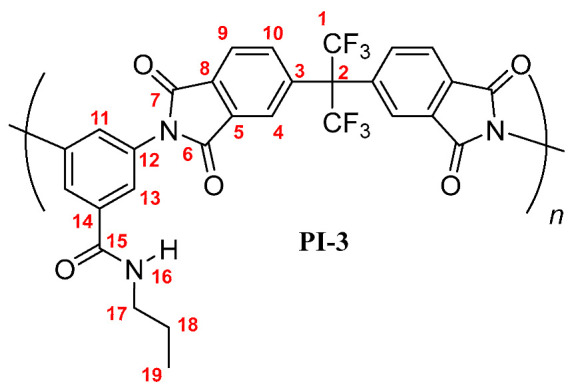


**PI-4:** FT-IR (KBr, ν, cm^−1^): 3403 (N–H, amide); 3084 (C–H, arom.); 2967, 2931, 2866 (C–H, aliph.); 1785 (C=O asym., imide); 1729 (C=O sym., imide); 1667 (C=O, amide); 1598, 1519, 1455 (C=C, arom. ring); 1356 (C–N, imide); 721 (imide ring deformation). ^1^H NMR (600 MHz, DMSO-*d_6_*, δ, ppm) 8.57 (broad peak, 1H, H-16); 8.20 (d, *J* = 7.9 Hz, 2H, H-9); 8.03 (s, 2H, H-4); 7.98 (broad peak, 2H, H-10); 7.78 (s, 2H, H-13); 7.74 (s, 1H, H-11); 3.27 (broad peak, 2H, H-17); 1.49 (m, 2H, H-18); 1.32 (m, 2H, H-19); 0.87 (t, *J* = 6.9 Hz, 3H, H-20). ^13^C NMR (150 MHz, DMSO-*d_6_*, δ, ppm) 166.75 (C-15); 166.22 (C-7); 165.97 (C-6); 138.02 (C-3); 136.50 (C-5); 136.40 (C-14); 133.79 (C-10); 133.25 (C-12); 133.12 (C-8); 129.07 (C-11); 126.81 (C-13); 124.91 (C-9); 124.33 (C-4); 124.01 (q, *J* = 286 Hz, C-1); 64.98 (hept, *J* = 25 Hz, C-2); 38.92 (C-17); 29.25 (C-18); 22.19 (C-19); 13.75 (C-20). Elem. Anal. Calcd. for [C_30_H_19_F_6_N_3_O_5_]*_n_* (615.49)*_n_*, C, 58.54%; H, 3.11%; N, 6.83%. Found: C, 57.98%; H, 2.99%; N, 6.27%.


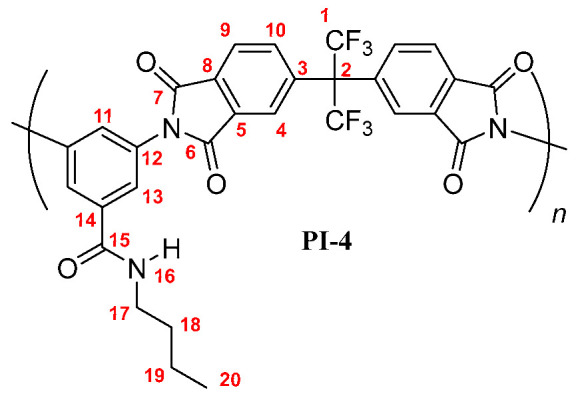


**PI-*t*-4:** FT-IR (KBr, ν, cm^−1^): 3409 (N–H, amide); 3090 (C–H, arom.); 2962, 2867 (C–H, aliph.); 1785 (C=O asym., imide); 1729 (C=O sym., imide); 1663 (C=O, amide); 1597, 1532, 1456 (C=C, arom. ring); 1355 (C–N, imide); 720 (imide ring deformation). ^1^H NMR (600 MHz, DMSO-*d_6_*, δ, ppm) 8.20 (d, *J =* 8.1 Hz Hz, 2H, H-9); 7.98 (m, 4H; H-4, H-10); 7.85 (broad peak, 1H, H-16); 7.77 (s, 2H, H-13); 7.70 (s, 1H, H-11); 1.36 (s, 9H, H-18). ^13^C NMR (150 MHz, DMSO-*d_6_*, δ, ppm) 166.88 (C-15); 166.25 (C-7); 165.98 (C-6); 138.06 (C-3); 136.41 (C-5); 136.50 (C-14); 133.86 (C-10); 133.32 (C-12); 133.19 (C-8); 129.18 (C-11); 126.73 (C-13); 124.89 (C-9); 124.40 (C-4); 123.89 (q, *J* = 286 Hz, C-1); 65.01 (hept, *J* = 25 Hz, C-2); 50.52 (C-17); 28.21 (C-18). Elem. Anal. Calcd. for [C_30_H_19_F_6_N_3_O_5_]*_n_* (615.49)*_n_*, C, 58.54%; H, 3.11%; N, 6.83%. Found: C, 58.03%; H, 3.19%; N, 6.73%.


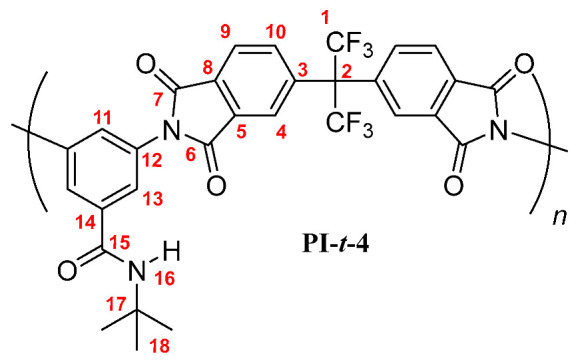


**PI-5:** FT-IR (KBr, ν, cm^−1^): 3403 (N–H, amide); 3087 (C–H, arom.); 2959, 2930, 2860 (C–H, aliph.); 1785 (C=O asym., imide); 1729 (C=O sym., imide); 1663 (C=O, amide); 1597, 1532, 1457 (C=C, arom. ring); 1355 (C–N, imide); 721 (imide ring deformation). ^1^H NMR (600 MHz, DMSO-*d_6_*, δ, ppm) 8.58 (broad peak, 1H, H-16); 8.20 (d, *J* = 7.9 Hz, 2H, H-9); 8.03 (s, 2H, H-4); 7.98 (broad peak, 2H, H-10); 7.77 (s, 2H, H-13); 7.74 (s, 1H, H-11); 3.26 (broad peak, 2H, H-17); 1.51 (broad peak, 2H, H-18); 1.28 (broad peak, 4H; H-19, H-20); 0.84 (broad peak, 3H, H-21). ^13^C NMR (150 MHz, DMSO-*d_6_*, δ, ppm) 166.87 (C-15); 166.30 (C-7); 166.15 (C-6); 138.02 (C-3); 136.48 (C-5); 136.48 (C-14); 133.79 (C-10); 133.35 (C-12); 133.21 (C-8); 129.17 (C-11); 126.97 (C-13); 124.96 (C-9); 124.21 (C-4); 123.84 (q, *J* = 286 Hz, C-1); 64.91 (hept, *J* = 25 Hz, C-2); 38.80 (C-17); 29.85 (C-19); 28.19 (C-18); 22.25 (C-20); 13.95 (C-21). Elem. Anal. Calcd. for [C_31_H_21_F_6_N_3_O_5_]*_n_* (629.52)*_n_*, C, 59.15%; H, 3.36%; N, 6.68%. Found: C, 58.99%; H, 3.03%; N, 6.52%.


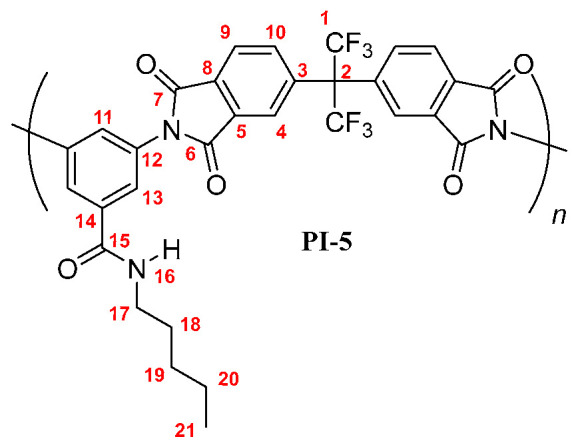


**PI-6:** FT-IR (KBr, ν, cm^−1^): 3407 (N–H, amide); 3088 (C–H, arom.); 2957, 2929, 2858 (C–H, aliph.); 1785 (C=O asym., imide); 1729 (C=O sym., imide); 1663 (C=O, amide); 1597, 1530, 1456 (C=C, arom. ring); 1354 (C-N, imide); 721 (imide ring deformation). ^1^H NMR (600 MHz, DMSO-*d_6_*, δ, ppm) 8.58 (broad peak, 1H, H-16); 8.20 (d, *J* = 7.8 Hz, 2H, H-9); 8.03 (s, 2H, H-4); 7.98 (broad peak, 2H, H-10); 7.77 (s, 2H, H-13); 7.74 (s, 1H, H-11); 3.26 (broad peak, 2H, H-17); 1.50 (m, 2H, H-18); 1.25 (m, 6H; H-19, H-20, H-21); 0.83 (broad peak, 3H, H-22). ^13^C NMR (150 MHz, DMSO-*d_6_*, δ, ppm) 166.86 (C-15); 166.19 (C-7); 166.09 (C-6); 138.02 (C-3); 136.49 (C-5); 136.46 (C-14); 133.80 (C-10); 133.35 (C-12); 133.21 (C-8); 129.18 (C-11); 126.99 (C-13); 124.97 (C-9); 124.22 (C-4); 123.85 (q, *J* = 286 Hz, C-1); 64.93 (hept, *J* = 25 Hz, C-2); 38.78 (C-17); 31.15 (C-20); 28.99 (C-18); 26.45 (C-19); 22.35 (C-21); 13.94 (C-22). Elem. Anal. Calcd. for [C_32_H_23_F_6_N_3_O_5_]*_n_* (643.54)*_n_*, C, 59.72%; H, 3.60%; N, 6.53%. Found: C, 59.21%; H, 3.43%; N, 6.38%.


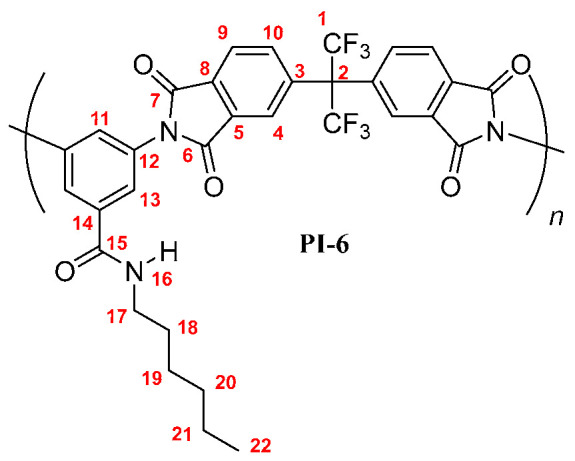


### 2.4. Film Preparation

Poly(imide) films were prepared by solution casting using the following procedure: Each polymer (480 mg) was dissolved at room temperature in THF (12 mL) and filtered through a 200 µm Teflon syringe filter and poured onto an aluminium ring placed on a glass plate. Then, the solvent was evaporated at room temperature for 24 h. After that, the films were immersed in deionized water, stripped off the plate, and dried in a vacuum oven at 190 °C for 24 h. The thickness of the membranes ranged from 40 to 60 μm.

### 2.5. Instrumentation and Measurements

FT-IR spectra (KBr pellets) were recorded on a Nicolet 8700 Thermo Scientific FTIR spectrophotometer over the range of 4000–450 cm^−1^. ^1^H and ^13^C NMR spectra for polymers were carried out on a 600 MHz instrument (Varian VNMRS) using DMSO-*d_6_* as solvent and TMS as internal standard, while ^1^H and ^13^C NMR spectra for monomers were recorded on a 400 MHz instrument (BRUKER AVANCE III HD-400). Viscosimetric measurements were made in an Ubbelohde viscosimeter number 50 at 30 °C (c = 0.5 g/dL). The size exclusion chromatography (SEC) measurements were performed on a GPC System 150cv (Santa Clara, CA, USA) at 20 °C equipped with a refractive index detector and a GPC KF-803 column (8.0 × 300 mm). The molecular weight (Mn and Mw) and polydispersity index were calculated according to the polyethylene glycol oxide standard and THF was used as a solvent. Soluble samples (c = 0.5 mg/mL) were filtered through micro-filters of 2 µm and then 100 µL were injected at 1 mL/min. Differential scanning calorimetry (DSC) was conducted on a TA Instruments Discovery DSC at a heating rate of 20 °C/min under nitrogen atmosphere. Thermogravimetric analysis (TGA) was performed using a thermogravimetric balance TGA-7 Perkin Elmer under a nitrogen atmosphere with a heating rate of 10 °C/min from 50 °C to 800 °C. Elemental analyses were made on a Fisons EA 1108-CHNS-O equipment. The mechanical properties of the films were measured with a Shimadzu AGS-X universal testing machine with a 100 N load cell at a strain rate of 1 mm/min, using strips of 5 mm wide, 50 mm long, and 40–60 µm thick, that were cut from polymer films. Wide-angle X-ray diffraction (XRD) was conducted on a Bruker D8 Advance diffractometer with CuKα radiation (wavelength *λ*_Cu_ = 1.542 Å), in range of 5° to 60° 2θ. The average *d*-spacing was calculated using Bragg’s law:

$$d=\frac{n\lambda }{2sin\theta }$$

where *θ* was assigned from the broad, amorphous peak maximum [20]. Poly(imide) density (ρ) was measured in a density gradient column (Techne Corporation, Minneapolis, MN, USA) with calcium nitrate solutions at 25 °C. Fractional free volume (FFV) was calculated by using the experimental density, and the theoretical volume occupied by the repeating unit of each polymer according to the following equation:

FFV = (V_sp_ − 1.3V_w-bondy_)/V_sp_

where V_sp_ is the specific volume (V_sp_ = ρ^−1^) and V_w-bondy_ is the Van der Waals volume occupied by the repeating unit of the polymer, which was calculated with Bondi’s group contribution method [21]. Pure gas permeability coefficients (*P*) were determined using a constant volume permeation cell of the type described elsewhere [22], according to the following equation:

$$P=\frac{273}{76}\frac{Vl}{ATp_{0}}\frac{dp}{dt}$$

where *A* and *l* are, the effective area and the thickness of the film, respectively. T is the temperature of the measurement (308.15 K), *V* is the constant volume of the permeation cell, *p_0_* is the pressure of the feed gas in the upstream, and *dp/dt* is the gas pressure increase with time under steady-state conditions measured in the permeation cell. *P* is expressed in Barrer [1 Barrer = 10^−10^ [cm^3^·(STP)·cm cm^−2^·s^−1^·cmHg^−1^]. The effective area of the film was 1.13 cm^2^. Before each permeation test, the film was degassed for 24 h. The pure gases evaluated were He, H_2_, O_2_, N_2_, CH_4_, and CO_2_, obtained from Praxair Corp. (San Salvador Xochimanca, Mexico) with purities > 99.99%. The measurements were carried out at 2 atm upstream pressure for each pure gas.

## 3. Results and Discussion

### 3.1. Monomers and Polymers: Synthesis and Characterization

The monomers synthesis started with the nucleophilic substitution reaction on the commercial acyl chloride (DNBC) using five primary amines as nucleophile agents. The 3,5-dinitro-*N*-alkylbenzamide derivatives were isolated as solids in good yields (90–95%). In the next step, the benzamide precursors were successfully reduced to the corresponding amide-diamine using a catalytic hydrogenolysis reaction (Yield: 87–93%). As it is known, one of the most critical factors to achieve high molecular weight polymers through polycondensation reactions is the purity of the monomers; therefore, all amide-diamine monomers and the commercial 6FDA were purified by sublimation technique before their use.

Monomers were structurally characterized by FT-IR and NMR techniques, as well as by elemental analysis (see Experimental Section). The FT-IR spectra of all monomers showed signals around 3400, 3330 and 1645 cm^−1^, associated with the symmetric and asymmetric stretching of the amino (NH_2_) and amide (NH and C=O) groups, respectively. Additionally, all the ^1^H-NMR and ^13^C-NMR signals were assigned for each monomer, where the most significant changes in NMR spectra were observed in the aliphatic region due to the different fragments [19].

The amide-diamine monomers were reacted with the commercial dianhydride 6FDA using anhydrous DMAc as solvent to obtain the aromatic PIs. In a first step, a viscous poly(amic acid) solution was obtained after 6 h of reaction, which underwent a chemical cyclization process with acetic anhydride and anhydrous pyridine as dehydrating agents. The fluorinated poly(imide)s were obtained in a 94–97% yield.

**Figure 1:**
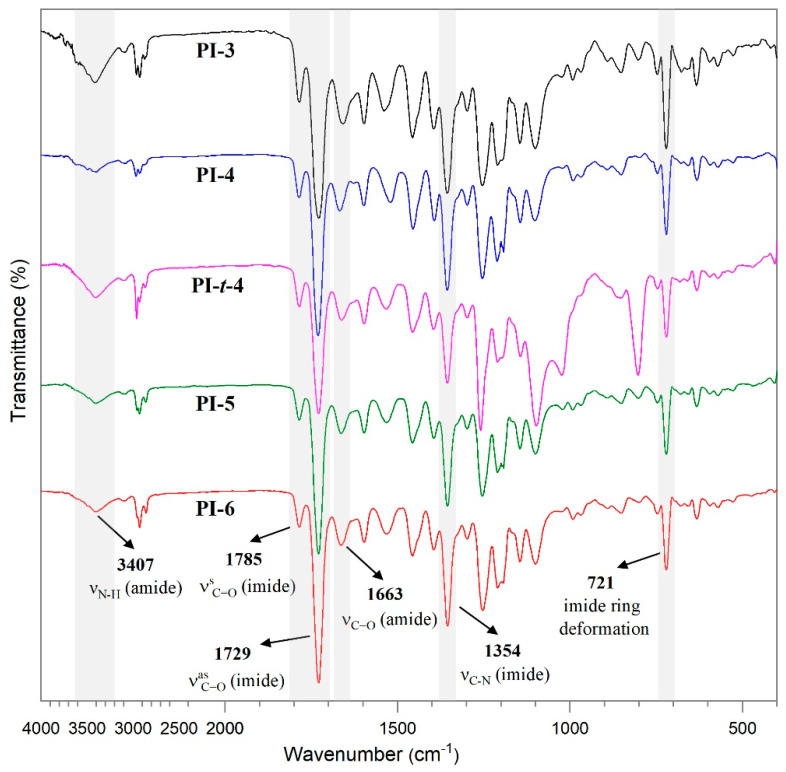
FT-IR spectra of the poly(imide)s.

The success of the polymerization reactions was confirmed by FT-IR spectroscopy. The FT-IR spectra of the poly(imide)s are shown in Figure 1. The peaks at 1785 and 1729 cm^−1^ were attributed to the characteristic symmetric and asymmetric C=O stretching of the imide rings, respectively. The signal around 1663 cm^−1^ indicates the C=O stretching of the amide groups. The peaks around 1354 and 721 cm^−1^ correspond to the C-N stretching and the imide rings deformation, respectively. Furthermore, the absorption bands about 3000 cm^−1^ were ascribed to the C-H stretching of the phenyl and alkyl groups, and the broad signal around 3407 cm^−1^ was assigned to the N-H stretching of the imide groups [19]. In addition, no bands associated to the bi-functionality of the used monomers were observed (N-H stretching for diamine at 3388 cm^-1^ or one of the stretching bands for anhydride (1853 cm^−1^).

**Figure 2:**
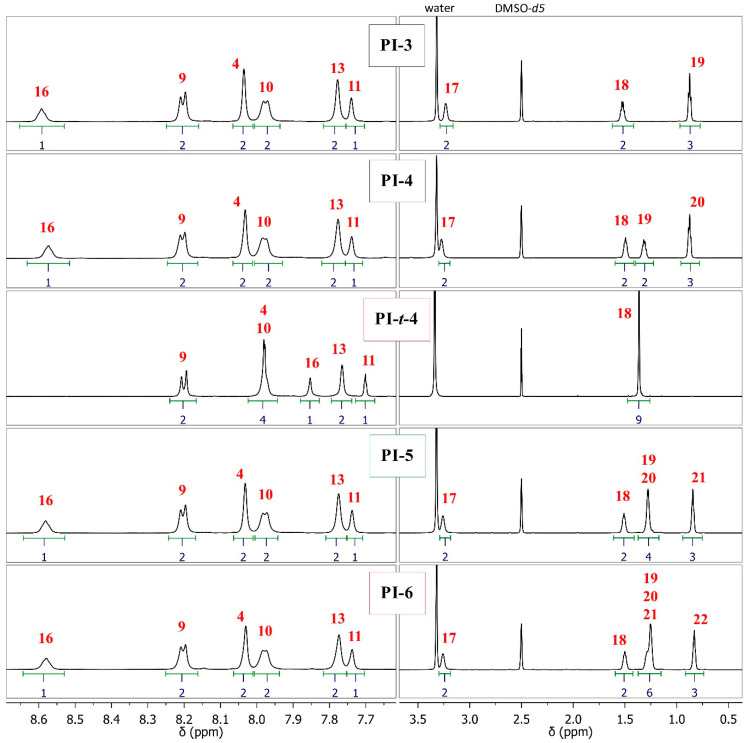
^1^H NMR spectra of all poly(imide)s.

The results of the NMR characterization also confirmed the chemical structure of each polymer [19,23]. Figure 2 shows the ^1^H-NMR spectrum for each poly(imide). In the aromatic region, protons from the dianhydride (H-4, H-9, and H-10) and the diamine monomers (H-11, H-13) were observed, evidencing the success of the polymerization reaction. The main difference among PIs spectra was the chemical shift of the PI-*t*-4 amide proton (H-16), which was more shielded than their homologue signals. This is mainly due to the steric-hindrance exerted by the *tert*-butyl group attached to the nitrogen atom. Another difference was the chemical shift of the proton H-11 also in the PI-*t*-4 proton spectrum. The slightly higher positive inductive effect of the *tert*-butyl group, compared to the inductive effect of the linear chains, decreases the negative mesomeric effect of the *N*-R-carbamoyl group on the proton H-11 in the *para* position. Therefore, the proton H-11 is slightly more shielded in PI-*t*-4 than in similar PIs. Moreover, the chemical shift and the integrated signal intensities in the aliphatic region of the ^1^H-NMR spectra were in accordance with the respective carbon chains of each poly(imide) [24].

### 3.2. Inherent Viscosity, Molecular Weight, and Solubility

Inherent viscosities were measured in a single point in NMP at 30 °C (c = 0.5 g/dL). The values ranged between 0.2 and 0.5 dL/g, indicating low to moderate molecular weights. Number (M_n_) and weight (M_w_) average molecular weights were measured by SEC. The values were in accordance with the results obtained for inherent viscosities, also indicating low to moderate molecular weights. Taking into account the molecular weights of their own repeating units, the chain ranged from 78–87 units long, except for PI-4, which was 45 units long. In fact, PI-4 exhibited the lowest inherent viscosity (0.2 dL/g) and molecular weight values in the series, leading to brittle thin film. It is not possible to offer a plausible explanation for the low molecular weight of this polymer, because M-4 were purified in the same way that other diamines, and the polymerization technique was the same one. Several attempts to synthetize PI-4, with special care in the monomers ratio (1:1) yielded the same result. Polydispersity indexes ranged from 1.3 to 1.5 and were within the range expected for condensation polymers.

Solubility is an important parameter for the processability of polymeric materials. In this sense, all poly(imide)s were soluble at 19 °C in a wide variety of aprotic polar organic solvents (Table 1) such as DMSO, NMP, DMF, and DMAc, as well as, THF, a relatively low boiling point solvent that favors their industrial processability. Additionally, PI-*t*-4 was soluble in chloroform. A possible interpretation of this outstanding solubility could be attributed to the bulky pendant groups (*tert*-butyl fragment) hanging along the polymer chain [15]. The *tert*-butyl groups increase the interchain distance, allowing the solvent to solvate the polymer chains more efficiently compared to the other PIs. As seen in Table 2, PI-*t*-4 had the highest fractional free volume (FFV) value, which corroborates the explanation for its excellent solubility.

### 3.3. Density, Fractional Free Volume, and Wide-Angle X-ray

The packing density of poly(imide)s was evaluated by determining the experimental density and theoretical FFV for each film (Table 2). The densities at 25 °C were in the range of 1.38–1.44 g/cm^3^. PI-*t*-4 showed the lowest experimental density and the highest FFV in the series, mainly due to the bulky *tert*-butyl groups along the poly(imide) structure, which reduce the packing efficiency of the chains. PI-6 had the lowest density, but the volume occupied by the repeating unit (V_w-bondy_) is the highest one, which reduces FFV.

**Figure 3:**
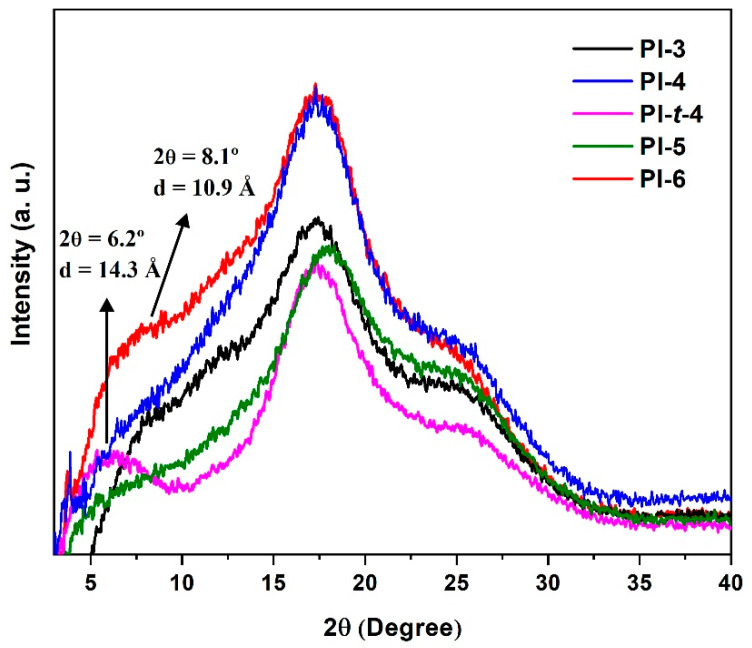
Wide-angle X-ray diffraction spectra of poly(imide)s.

Figure 3 shows the wide-angle X-ray diffraction profiles for poly(imide)s. A wide halo is observed in all spectra, which indicates that the samples are amorphous. PI-*t*-4 produced two peaks of 2θ angle at 6.2° and 17.8°, while PI-6 exhibited a little shoulder in 2θ at 8.1° and a maximum at 17.8°. The remaining poly(imide)s only showed one maximum. According to previous works, the interchain distance could be calculated through Bragg’s equation, based on the position of the amorphous halo maximum [20]. PI-*t*-4 showed the highest interchain distance, with angles of 14.3 and 5.0 Å, respectively. This suggests that the introduction of *tert*-butyl moieties as pendant groups produces looser packing compared to the other *n*-alkyl pendant groups. PI-6, with six carbon atoms in the pendant chain, also showed two *d*-spacing values with an interchain distance of 10.9 and 5.1 Å.

In our previous work, with cycloalkyl pendant groups (cyclopentyl, cyclohexyl, cyclooctyl and adamantly fragments) attached to the main chain, the *d*-spacing values were between 6.1 and 6.4 Å, which was indicative of moderate to high interchain distances [19]. However, none of those polymers showed two maximum halos in the X-ray pattern. Probably, PIs with acyclic alkyl pendant groups stack differently compared to cyclic side groups PIs in amorphous solid state, by virtue to their flexible nature. This packing would have created regions with greater interchain distance than others. The rigid nature of the cyclic fragments used in our previous work prevented this behaviour.

### 3.4. Thermal and Mechanical Properties

**Figure 4:**
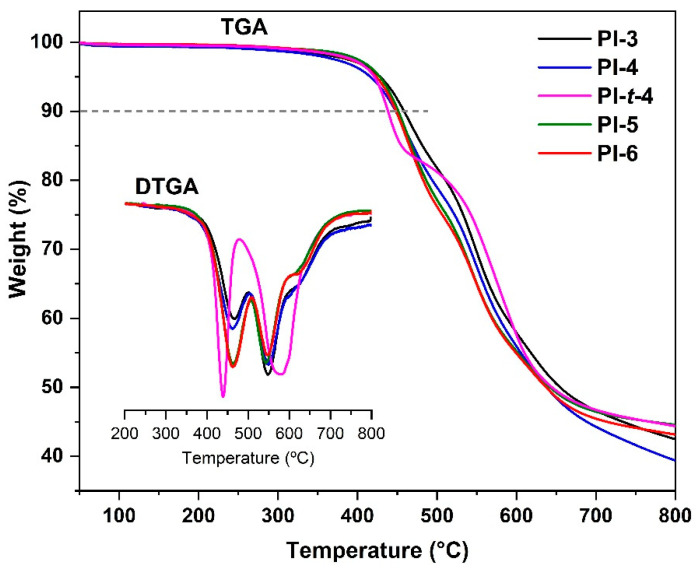
TGA and DTGA curves of poly(imide)s.

The thermal properties of the poly(imide)s were determined by thermogravimetric analysis (TGA) and differential scanning calorimetry (DSC) under the nitrogen atmosphere. The TGA and DTGA curves of all poly(imide)s are shown in Figure 4, while the char yield and temperature at 5% and 10% weight loss values are summarized in Table 3.

All poly(imide)s were highly thermally stable, with onset degradation temperature up to 415 °C. The thermograms had two well-defined stages of weight loss, as shown in the derivative curve (DTGA). The first stage, centered between 445 °C and 458 °C, is related to the decomposition of alkyl chains, while the second stage (550–580 °C) is attributed to the rupture of bonds in the poly(imide) backbone [18,25,26].

**Figure 5:**
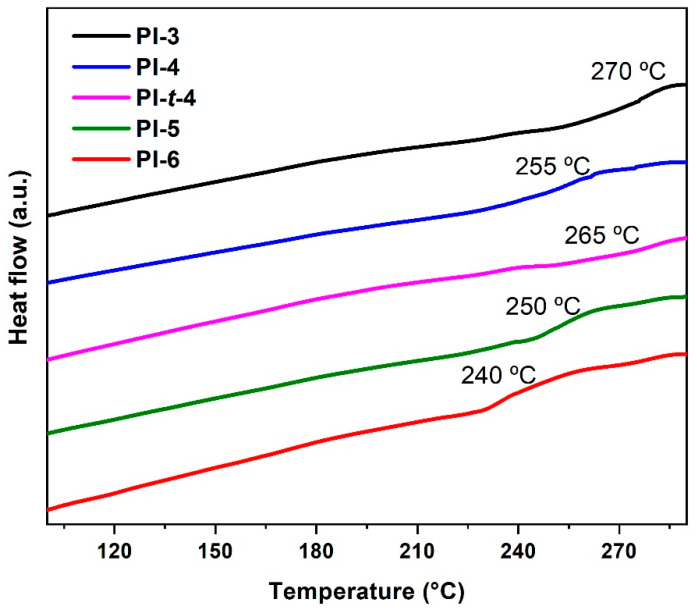
DSC PIcurves of poly(imide)s.

The DSC curves are presented in Figure 5, and the glass transition temperature (T_g_) values are summarized in Table 3. T_g_ values of the poly(imide)s were in the range of 240–270 °C. In a polymer chain, the degrees of freedom increase with the number of carbon atoms, which leads to more flexible chains and, therefore, to T_g_s [27,28,29]. In this sense, a decrease in T_g_ values was observed with the increase in the size lateral groups, from propyl to hexyl fragments. Because of its bulky nature, PI-*t*-4 does not follow this trend and gives a PI with higher rigidity and increased T_g_.

The mechanical properties values of the PI films are given in Table 4. The films had a tensile strength in the range of 45–87 MPa, elongation at break in the range of 3–11%, and tensile modulus in the range of 1.4–2.0 GPa. It was not possible to measure the mechanical properties of PI-4 film as it was brittle, which is attributed to its low molecular weight.

It is expected that, for a same PIs backbone with different pendant acyclic alkyl chains, the Young’s modulus increases with the length of these. The larger the pendant acyclic alkyl chains, the higher the cross-interactions between polymer backbones and the harder their mobility (increases the stiffness). This behaviour is observed when Young’s Modulus of PI-3 is compared with those in PI-5 or PI-6. In the case of PI-*t*-4, the bulkiness of the *tert*-butyl moiety hinders the extension of mobility between PIs chains and therefore, a higher value is observed in the Young’s modulus.

On the other hand, the observed increase in tensile strength from PI-3 to PI-6 can be attributed to the increase in molecular weight [30,31]. For similar structures, the higher the molecular weight, the stronger the intermolecular interactions and therefore the strength of the material. As can be noted, the elongation at breaks follows the same trend as in tensile strength except for PI-*t*-4, which showed a lower value (3.2%). This lower value could be explained considering that the mechanism of the plastic deformation is the sliding between the polymer chains in the material. The bulkiness of *tert*-butyl moiety in PI-*t*-4 do not favour the long-range interactions between the polymer chains as the linear alkyl moieties do. These long-range interactions are in charge of extending the elongation before the material breaks. Moreover, as side chains become lengthier, there are more possibilities of entangling the polymer chain, resulting in higher tensile strength. However, at the same time, the side chain becomes more flexible, which improves the elongation at break [32,33].

### 3.5. Gas Transport Properties

The effect of the acyclic alkyl-*N*-carbamoyl groups on the pure gas transport properties of poly(imide)s was evaluated, using a constant volume permeation cell at 2 atm and 35 °C. Table 5 shows the pure gas permeability coefficient for He, H_2_, O_2_, N_2_, CH_4,_ and CO_2,_ gases and the ideal gas selectivity values for O_2_/N_2_, CO_2_/CH_4,_ and the CO_2_/N_2_ gas pair. Since it was not possible to prepare an adequate film for PI-4, these properties could not be measured for this polymer. The permeability coefficient order for the remaining poly(imide)s was *P*He > *P*H_2_ > *P*CO_2_ > *P*O_2_ > *P*N_2_ > *P*CH_4_, which followed the same order as the gas kinetic diameter for these six gases (He 2.6 Å, H_2_ 2.89 Å, CO_2_ 3.3 Å, O_2_ 3.46 Å, N_2_ 3.64 Å, CH_4_ 3.8 Å) [34,35]. These results indicate that gas diffusion process plays a vital role in gas permeation through these polymers.

PI-*t*-4 showed the highest permeability and selectivity for all gases, which is attributed to the presence of the bulky *tert*-butyl pendant groups along the polymer chain [36]. Regarding the other poly(imide)s, the permeability for all gases decreased as follows: PI-6 > PI-5 > PI-3 (as the alkyl chains are shortened). In general, these results correlate with the calculated FFV and the obtained *d*-spacing values. Interestingly, permeability of PI-*t*-4 film was 2-3 times higher compared to PIs bearing acyclic alkyl groups films for all gases. PI-t-4 was actually more permeable than the polymers containing cyclic pendant groups, previously synthesized by our group [19]. Even PI-6 displayed a permeability for CO_2_ (*P*CO_2_ = 32 Barrer) similar to the homologous poly(imide) having an adamantyl pendant group (*P*CO_2_ = 31.8 Barrer). The acyclic alkyl chains are probably less resistant to the pressure from gases, allowing them to diffuse more easily through the film. However, the selectivity of these poly(imide)s decreased as the gas permeability increased. Such behaviour has already been reported in previous works [19,37]. In Table 5, data available for Matrimid, a commercial poly(imide) membrane, was incorporated for comparison [3]. All PIs prepared in this work were more permeable than Matrimid, but Matrimid was more selective, as expected according to the trade-off between permeability and selectivity reported by Robeson [38,39].

## 4. Conclusions

Five new aromatic poly(imide)s with acyclic alkyl pendant groups were successfully synthesized by polycondensation reaction in solution, and structurally characterized. In general, PIs exhibited moderate molecular weights, were soluble in aprotic polar solvents, were thermally stable up to 415 °C, and had T_g_ values in the range of 240–270 °C. Mechanical properties were also measured, except for PI-4, giving values of tensile strength, elongation at break, and tensile modulus in the range of 45–87 MPa, 3–11%, and 1.4–2.0 GPa, respectively. The poly(imide) containing the most branched substituent (PI-*t*-4, *tert*-butyl fragment) was the most soluble and most permeable to gases. Those results are in agreement with the largest fractional free volume recorded for its film. The longest interchain distance in PI-*t*-4 was corroborated indirectly through density measurements, and directly by means of wide-range X-ray diffraction. The structure-property relationship for these new poly(imide)s is a crucial point to take into account because the results presented here demonstrated that the incorporation of *tert*-butyl-*N*-carbamoyl moieties along the polymer chain leads to films with increased permeability to different gases compared to previously reported PIs films bearing cyclic bulky groups.

## Figures and Tables

**Table 1:** Inherent viscosity, molecular weight, and solubility.

PIs	η_inh_^a^ (dL/g)	Mn (×10^4^ Da)	Mw (×10^4^ Da)	DPI	Solubility at 19 °C					
					DMSO	DMF	DMAc	NMP	THF	CHCl_3_
PI-3	0.3	4.728	6.321	1.3	+	+	+	+	+	-
PI-4	0.2	2.793	4.176	1.5	+	+	+	+	+	-
PI-*t*-4	0.4	5.385	7.364	1.4	+	+	+	+	+	+
PI-5	0.4	5.287	6.976	1.3	+	+	+	+	+	-
PI-6	0.5	5.897	8.131	1.4	+	+	+	+	+	-

^a^ Measured in NMP (0.5 g/dL at 30 °C). DPI: polydispersity index. Solubility: +, Soluble; -, Insoluble.

**Table 2:** Density, fractional free volume, and *d*-spacing.

PIs	Ρ ^a^ (g/cm^3^)	V_w-bondy_^b^ (cm^3^/mol)	FFV ^c^	Dsp ^d^ (Å)
PI-3	1.4423	278.23	0.132	5.0
PI-4	n.d.	n.d.	n.d.	5.1
PI-*t*-4	1.3806	288.45	0.158	5.1; 14.3
PI-5	1.3982	298.69	0.137	4.9
PI-6	1.3803	308.92	0.138	5.1; 10.9

^a^ Density: measured at 25 °C. ^b^ Van der Waals volume calculated by Bondi’s group contribution method [21]. ^c^ Fractional Free Volume. ^d^ *d*-spacing estimated by DRX.

**Table 3:** Thermal properties of poly(imide)s.

PIs	T_5_ (°C) ^a^	T_10_ (°C) ^b^	Char Yield (%)	T_g_ (°C) ^c^
PI-3	428	458	42	270
PI-4	416	448	39	255
PI-*t*-4	421	455	44	265
PI-5	428	468	45	250
PI-6	423	448	43	240

^a,b^ Thermal decomposition temperature at which 5% and 10% weight loss, respectively. ^c^ Glass transition temperature (taken from the second heating scan).

**Table 4:** Mechanical properties of PIs.

PIs	Tensile Strength (MPa)	Elongation at Break (%)	Young’s Modulus (GPa)
PI-3	45	4.10	1.43
PI-*t*-4	53	3.2	2.19
PI-5	72	5.28	1.94
PI-6	87	11.98	1.74

Each sample was analyzed in triplicate. PI-4 film was too brittle to be measured.

**Table 5:** Pure gas permeabilities and ideal selectivity for poly(imide)s.

PIs	Permeability (Barrer ^a^)						Ideal Selectivity (α)		
	He	H_2_	O_2_	N_2_	CH_4_	CO_2_	O_2_/N_2_	CO_2_/CH_4_	CO_2_/N_2_
PI-3	52	46	5.4	1.2	1.1	23	4.5	21.3	19.9
PI-*t*-4	139	136	16.7	3.3	2.3	75	5.1	32.1	22.7
PI-5	44	42	5.9	1.4	1.2	27	4.2	21.9	19.8
PI-6	45	43	6.7	1.7	1.7	32	3.9	18.9	19.5
Matrimid *	-	-	2.1	0.3	0.3	10	6.5	35.7	31.2

^a^ 1 Barrer = 10^−10^ [cm^3^·(STP)·cm cm^−2^·s^−1^·cmHg^−1^. * Data reported in Ref. [3].
